# Confronting a New Challenge in Plastic Surgery: MDR Infections in Patients with Chronic Wounds

**DOI:** 10.3390/life14040444

**Published:** 2024-03-27

**Authors:** Laura Răducu, Oriana Elena Moraru, Daniela-Elena Gheoca-Mutu, Teodora Peligrad, Andrada-Elena Țigăran, Abdalah Abu-Baker, Daniela-Elena Ion, Bogdan Mihai Ursuț, Cristian-Radu Jecan, Adelaida Avino

**Affiliations:** 1 Discipline of Plastic Surgery, “Carol Davila” University of Medicine and Pharmacy, 020021 Bucharest, Romania; laura.raducu@umfcd.ro (L.R.); cristian.jecan@umfcd.ro (C.-R.J.); adelaida.avino@drd.umfcd.ro (A.A.); 2 Department of Plastic and Reconstructive Surgery, “Prof. Dr. Agrippa Ionescu” Emergency Clinical Hospital, 011356 Bucharest, Romania; daniela-elena.mutu@umfcd.ro (D.-E.G.-M.); andrada-elena.tigaran@rez.umfcd.ro (A.-E.Ț.); abdalah.abu-baker@drd.umfcd.ro (A.A.-B.); daniela-elena.ion@rez.umfcd.ro (D.-E.I.); 3 Discipline of Cardiovascular Surgery, “Carol Davila” University of Medicine and Pharmacy, 020021 Bucharest, Romania; 4 Department of Vascular Surgery, “Prof. Dr. Agrippa Ionescu” Emergency Clinical Hospital, 011356 Bucharest, Romania; 5 Discipline of Anatomy, “Carol Davila” University of Medicine and Pharmacy, 020021 Bucharest, Romania; bogdan.ursut@umfcd.ro; 6 Doctoral School, “Carol Davila” University of Medicine and Pharmacy, 020021 Bucharest, Romania; 7 Department of General Surgery, “Prof. Dr. Agrippa Ionescu” Emergency Clinical Hospital, 011356 Bucharest, Romania

**Keywords:** MDR infections, chronic wounds, ulcers, diabetes, diabetic foot ulcers

## Abstract

Background: The presence of a wound can be anywhere from non-problematic to life-threatening on a severity spectrum, with bacterial infection and resistance playing a major role in the development of chronicity, delaying wound healing. Wound colonization with multiple organisms and the limited number of effective antibiotics place a heavy burden on the healthcare system, with patients going through multiple surgeries during a prolonged hospitalization time. By analyzing the resistance patterns of pluri-bacterial populations and the approach used in managing complex cases, we aim to improve the protocols applied in caring for chronic wounds in our practice and share our experiences and observations. Methods: We designed a retrospective study on 212 diabetic and non-diabetic patients, aiming to evaluate the course of chronic wound treatment in our practice. We focused on the impact that MDR bacteria and diabetes have on surgical outcomes and their role in the healing process. Results: Patients who received empiric antibiotic therapy before being admitted eventually presented with multiple MDR bacteria compared to those who did not receive antibiotics (*p* = 0.014). The presence of at least one MDR bacteria in the wound bed was associated with ulcers reaching bone (*p* = 0.02) and was positively correlated with the number of surgeries performed (*p* < 0.001). Diabetes played a significant role in surgery-related complications (*p* = 0.02) and hospitalization time (*p* < 0.001). Conclusions: Proper management of chronic wounds requires a comprehensive, multidisciplinary approach and a thorough understanding of antibiotic usage. To address this need, we have developed and implemented a chronic wound treatment protocol in our clinic, with the goal of discharging patients once their ulcers have been treated and closed. A key summary of the protocol presented is to reduce the incidence of MDR bacteria and improve the patient’s quality of life.

## 1. Introduction

The skin’s main barrier function means permanently adapting to environmental and metabolic changes. This implies that any injury triggers a cascade of processes that aim to aid its recovery. When the protective barrier provided by the skin loses its main functions and exposes the rest of the system to pathogens, the wound-healing process becomes essential [1]. Wound repair can be affected by many factors, ranging from those concerning the host—the patient’s immunological status, age, genetic conditions, and comorbidities—to the skin microbiota and the pathogens involved [2]. 

The healing process can differ depending on the type of wound, whether it is acute or chronic. A wound is considered chronic if it does not follow a timely and orderly manner of repair and typically lasts more than four weeks. Moreover, chronic wounds are usually stuck in the inflammatory phase and are rich in modified neutrophils [3]. 

Despite differences in etiology, chronic wounds share certain features. These include excessive levels of proinflammatory cytokines, persistent infection, the formation of drug-resistant microbial films, and senescent fibroblasts with diminished migratory capacity. Furthermore, chronic wounds are often unresponsive to growth factor signals [4].

These types of wounds bring with them a high probability of infection. The infection in chronic wounds is frequently polymicrobial, aerobic, and anaerobic, boosting synergistic effects [5]. Moreover, many pathogens colonizing chronic wounds are antibiotic-resistant. Infections by antibiotic-resistant bacteria are responsible for around 700,000 deaths per year worldwide and are estimated to be accountable for over 10 million deaths per year in 2050 [6]. It is also important to acknowledge that the IDSA (Infectious Disease Society of America) has highlighted a new group of pathogens capable of escaping the biocidal actions of antibiotics—acronymically called ESKAPE. The bacteria included in the group are *Enterococcus faecium*, *Staphylococcus aureus*, *Klebsiella pneumoniae*, *Acinetobacter baumanii*, *Pseudomonas aeruginosa*, and *Enterobacter* spp. Most of these pathogens are multidrug-resistant (MDR) [7]. 

MDR means resistance to more than three different antibiotic classes. Characterized by potential drug resistance mechanisms, these bacteria may cause life-threatening infections among immunocompromised or critically ill patients, especially diabetics [8,9]. This is because diabetic patients have a higher risk of developing chronic ulcers that lower the quality of life and have a significant impact on the healthcare system [10]. Around one in four diabetic patients will develop a foot ulcer in their lifetime, with more than half of diabetic foot ulcers (DFU) becoming infected [11]. Prevention of DFU is crucial, as the risk of amputation for moderate to severe DFU is approximately 20%, and the mortality after diabetes-related amputation exceeds 70% at five years [12]. Although 77% of diabetic ulcers heal within the first year, the recurrence rate is 40% within the first year of recovery and 65% within five years. It is best to refer to these patients as being in remission rather than completely healed [13]. 

MDR infections are present in up to 53% of patients with DFUs and are linked to larger ulcer size, more severe ulcer classification, osteomyelitis, a previous history of antibiotic therapy, and hospitalization [14,15]. The hostile conditions of MDR bacteria can lead to a probability of progressing to complications, for instance, lower limb amputation [16]. 

According to the World Health Organization, one of the biggest threats to global health is antimicrobial resistance, with a continuous decline in the number of effective molecules against bacteria [17,18]. 

Successful treatment of infected chronic wounds often requires multidisciplinary management, including attention to wound care, revascularization, correction of metabolic abnormalities, antimicrobial therapy, and surgery [19]. This paper aims to emphasize the need for thorough protocols for dealing with MDR bacteria in chronic wounds, with a focus on diabetic patients. Thus, this study aims to analyze the protocol used at the Plastic Surgery Department of the Clinical Emergency Hospital “Prof. Dr. Agrippa Ionescu” in Bucharest, Romania. 

## 2. Materials and Methods

The purpose of this paper is to analyze and explain the protocol for chronic wound care while also discussing the impact of MDR bacteria and diabetes on chronic ulcers. 

Our team believes that proper management of chronic wounds requires a comprehensive, multidisciplinary approach and a thorough understanding of antibiotic usage. To address this need, we have developed and implemented a chronic wound treatment protocol in our clinic, with the goal of discharging patients once their ulcers have been treated and closed. By doing so, we aim to reduce the incidence of community-acquired infections and the selection of MDR bacteria in patients with chronic ulcers.

**Figure 1:**
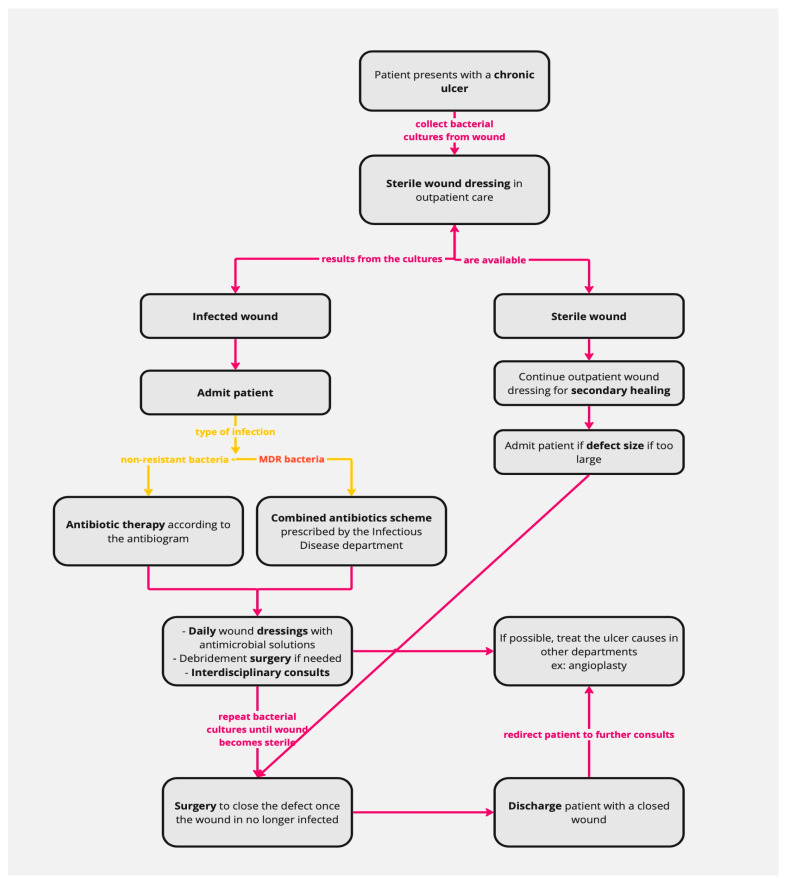
Protocol scheme. Pink arrows and writing—steps taken in patient management. Yellow arrows and writing—dividing patient lot based on type of infection. Red writing—MDR bacteria detected.

The protocol for chronic wounds implemented in our hospital (Figure 1) begins with collecting bacterial cultures from all patients with chronic wounds. We decided to stop treating ulcers with empiric antibiotics because this can increase the number of resistant pathogens. Local antiseptic dressings are applied to the wounds until the antibiogram results are received. 

All the patients are admitted when the results are available and are prescribed in-hospital antibiotic treatment accordingly. If MRD bacteria are detected, the patient follows a combined antibiotic scheme prescribed by the Infectious Disease department. Moreover, bacterial cultures are collected during the patient’s hospital stay to assess their progress. After hospital admission, the ulcers are dressed daily using special antimicrobial solutions that help decrease the microbial load. In order to ensure the best possible outcomes, we utilize a range of dressings that are specifically selected based on each patient’s individual needs. Examples of these novel materials used are silver nanoparticle-impregnated dressings, occlusive dressings impregnated with petrolatum and bismuth tribromophenate, and, if indicated, negative-pressure wound therapy. Surgery is then performed depending on the case (surgical debridement and amputation).

The general status of the patient is evaluated multidisciplinaryly on a case-by-case basis (e.g., in the Vascular Surgery department, Diabetes and Nutrition department, Cardiology department, etc.), and further treatment is guided by these recommendations. We also aim to treat the cause of the ulcers when possible. For example, in the case of vascular ulcers, the patient is transferred to the Vascular Surgery department for angioplasty.

Closure of the wound is only performed on a sterile wound, using various methods such as direct sutures, grafts, and flaps. The patients are discharged when they have a closed wound with no infection or complications. After discharge, the patients are redirected to specialty consultations to prevent the appearance of new ulcers. This protocol aims to limit the selection of MDR bacteria by treating them with a multidisciplinary approach and facilitating the closure of chronic wounds.

This observational retrospective study examines 212 patients admitted to the Plastic Surgery Department of the Clinical Emergency Hospital “Prof. Dr. Agrippa Ionescu”, Bucharest, Romania, for various chronic wounds in 2021 and 2022. All the patients were treated following our clinic’s protocol for chronic wounds. We aim to analyze the efficacy of our protocol.

In our study, we included a broad spectrum of chronic wounds. The exclusion criteria were children, pregnant women, and patients who did not agree to this study. All the patients included in this study were over 18 years old and agreed to participate, having signed an informed consent form.

Age, gender, diabetic status, duration of hospital stay, number of surgical procedures, ulcer type and characteristics, antibiotic treatment prior to culture sampling, and complications were all noted. From the bacterial cultures, data regarding the pathogens, the number of MDR bacteria, and antibiotic resistance were collected. We obtained information about the patient’s antibiotic intake before hospital admission, the use of medical devices in the last three months (urinary catheters, arterial/venous catheters, recipient of blood transfusion, and history of Clostridium diff. infection) to assess the risk of infection during the hospital stay.

Statistical analysis was performed using Chi-square tests and Pearson correlation, with the help of IBM SPSS v29.0. The manuscript was redacted using Microsoft Word 2021.

## 3. Results

The patient sample consisted of 212 patients, 106 of whom were admitted in 2021 and 106 of whom were admitted in 2022. The data were collected from our clinic database. 

We deliberately selected our patient cohort from 2021 and 2022 for this study’s design. This strategic choice was underpinned by the unique and unprecedented circumstances of the 2020 year, marked by the COVID-19 pandemic. The epidemiological context of this imposed significant restrictions on patient mobility and access to healthcare facilities. Consequently, this period of reduced medical services might have led to an aggravation of chronic wounds and their complications, as well as a higher incidence of infections.

The mean age in our sample was 59 years, and the median was 62 years. We identified a male predominance in our sample: 66% (140 patients) of the patients included in our study were males, while 72 were females. 

Further analyzing the pathogens involved, the data from the bacterial cultures showed a total of 64.2% MDR bacteria (resistant to more than three different antibiotic classes), 70.7% in 2021, and 57.5% in 2022, noticing a decrease over the years.

Eighty-three patients (38.7%) were diabetics, 66 male patients and 16 women. We used a Chi-square test to determine a statistically significant correlation between male patients and diabetes (*p* < 0.001; likelihood ratio 13.03).

However, when trying to correlate the MDR infections with the diabetic patients, we did not find any statistically significant correlations. Given that out of the 212 patient cohort, only 56 patients presented with both MDR and diabetes, we expected this result.

We decided to analyze the two risk factors—MDR and diabetes—separately and to assess their interference with the evolution of chronic ulcers. 

We identified a total of 255 ESKAPE pathogens, including *Enterococcus faecium* (7, or 3.3%), *Staphylococcus aureus* (135, or 63.68%), *Klebsiella pneumoniae* (33, or 15.56%), *Acinetobacter baumanii* (8, or 3.77%), *Pseudomonas aeruginosa* (51, or 24.05%), and *Enterobacter* spp. (21, or 9.90%). Further analyzing the results of the antibiograms, we noticed a high antibiotic resistance, as shown in Table 1. 

A positive correlation was found using a Chi-square test regarding patients who received antibiotic therapy before admission (*p* = 0.035; likelihood ratio 4.49; Table 2). Patients who took antibiotics before being admitted presented with MDR infections more frequently. This is one of the results that support our protocol.

Surgical treatment was part of our protocol. Therefore, surgeries were performed for the chronic defects: debridement and preparation of a clean recipient area if skin grafting or flap surgery was required, or direct suture if the size of the tissue loss allowed for proper wound closure. The number of surgical procedures performed was included in the database. A statistically significant positive correlation was established between the presence of MDR bacteria and the number of surgical interventions (*T*-test: *p* < 0.001; mean difference −0.48; 95% confidence interval for the difference (−0.78; −0.19)). We also observed a higher frequency of wound dehiscence and graft rejection in the MDR-positive patients’ sample. MDR-infected patients needed more surgical procedures and had more complications. 

Using a Pearson correlation test, we determined a positive relationship between the number of MDR bacteria, hospitalization time (*p* < 0.001; 95% confidence interval for the difference (0.35; 0.56)), and the number of surgeries performed (*p* < 0.001; 95% confidence interval for the difference (0.22; 0.46); Table 3). Patients who tested positive for MDR bacteria spent an average of 25 days in the hospital, compared with 10 days for patients with no MDR bacteria identified in their cultures. This is another argument that supports our protocol.

In addition, the ulcers were analyzed by assessing the depth of the tissue damage: (1) the skin defect reached the dermal layer, (2) the supra-fascial ulcer, and (3) the ulcer reached muscle/bone. We correlated the depth of the tissue damage with the presence of MDR bacteria. Using a Chi-square test, a positive correlation was observed between wounds infected with MDR bacteria and grade 3 ulcers. This result shows that an MDR-infected ulcer has a higher risk of more tissue damage (*p* = 0.02, likelihood ratio = 8.4).

In the case of diabetic patients, a higher number of surgeries was required for the proper healing of diabetic wounds (*t*-test; *p* < 0.001; mean difference −0.67; 95% confidence interval for the difference (−0.05; −0.289)). 

An increased rate of complications, such as wound dehiscence and skin graft rejection/flap failure, was observed in patients with diabetes (*p* = 0.02; likelihood ratio 5.17). This contributed to the higher number of surgeries needed and more extended hospital stays. Only 11 out of 117 non-diabetic patients suffered complications, compared to 16 out of 82 diabetic patients. This led to longer hospitalization time, an average of 30 days compared with the average of 13 days of hospitalization in the non-diabetic patients’ group (*T*-test; *p* < 0.001; mean difference −17.41; 95% confidence interval for the difference (−25.40; −9.41)). 

Furthermore, data regarding the wound site of every patient was split into four categories: digits, foot, calf, and others. Using a Chi-square test, we established a positive correlation between the digits and foot ulcer site and diabetic patients (*p* < 0.001), compared to the non-diabetic group, which did not show a statistically significant correlation with any wound site in particular (Table 4). 

## 4. Discussion

Nowadays, raising awareness about patients with chronic ulcers and finding the proper treatment for them is challenging for the healthcare system. These patients need extensive care, are hospitalized for long periods, and require large amounts of resources. We decided to implement a protocol for these patients to manage their cases better. The need for such protocols comes from a rising number of MDR bacteria colonizing chronic wounds and complications of DFU. It is essential to acknowledge and declare the presence of MDR bacteria while trying to treat them. This can only be achieved if a multidisciplinary approach is followed. 

Having said that, one of the first and most important factors we presented in this manuscript was MDR infections in patients admitted to our clinic. The results showed a total of 64.2% MDR bacteria and a decrease over the two years.

Considering the differences between 2021 (70.7%) and 2022 (57.5%), there was a noticeable decrease. In structuring our research, we consciously chose to commence our patient selection process starting in 2021. This decision was primarily influenced by the extraordinary circumstances that defined 2020. The COVID-19 pandemic brought forth extensive restrictions and public health measures, significantly altering the accessibility and use of medical services. Shin et al., in their study “A Tale of Two Cities”, reported a nearly 50% decrease in foot clinic visits in Manchester (United Kingdom) and almost a 70% drop in Los Angeles (U.S.) after lockdown [20]. Another study by Guest et al. in the United Kingdom also showed a >50% reduction in face-to-face clinician visits in 2020 and 2021 for patients with venous leg ulcers, with repercussions on the healing rates [21]. It is well known that proper adherence to the CDC (Centre for Disease Control and Prevention) guidelines for surveillance, such as hand hygiene and disinfection of hospital surfaces, is critical to controlling MDR organisms [22]. In addition, our hospital policies and protocols changed in 2021 with the implementation of stricter epidemiological control and the protocol for chronic ulcers. Therefore, we expect to see a high percentage of infected wounds in 2021, followed by a decrease in 2022. We believe this direction can be followed in developing future studies.

Even though 57.5% can still be considered a high percentage, the rising presence of ESKAPE pathogens and their increased antibiotic resistance must be considered. A systematic review of the clinical and economic impact of antibiotic resistance found ESKAPE to be associated with the highest risk of mortality [23]. Boucher et al. state that for the ESKAPE pathogen’s treatment, the available efficient drugs must be protected, hence the need for better hospital infection control practices [24]. The number of drugs effective against ESKAPE is declining, predisposing us toward a future without effective antibiotics. A review by Mulani et al. discusses the importance of understanding antibiotic action and how to properly use them in combination for ESKAPE, while emphasizing the need for new therapies [25].

This study shows a statistically significant correlation between patients who took antibiotics before hospital admission and the subsequent development of MDR bacteria. Out of the 90 patients who received antibiotics before hospital admission, 65 (72.22%) presented with MDR infections. Our protocol for chronic wound management includes using antibiotics following the antibiogram and only during hospital admission, aiming to prevent the selection of MDR bacteria. There are multiple reports of similar trends, talking about how resistant bacteria can be selected due to the misuse of antibiotics. Baran et al. affirm that the overprescription and widespread availability of antibiotics lead to a growing number of multidrug-resistant microbes [26].

This finding underscores the potential utility of risk assessment tools, such as the Carmeli score, in managing infectious wounds. A comprehensive patient history, including the previous antibiotic intake, can be proven to be an instrumental tool in tailoring individual treatment strategies, therefore increasing efficacy in wound management. In a review, Vandenbroucke-Grauls and Schultsz point out that surveillance and risk-assessing strategies can be helpful tools for infection control and strategy evaluation. An interesting point is made about prospective surveillance. Over four years after implementing the NNIS System (National Nosocomial Infections Surveillance System), the rates of nosocomial infections decreased steadily [27]. This also supports our statement that a protocol is needed to treat such wounds. The reason for starting this study is to show how applying our protocol can help decrease the MDR presence and the DFU complications. 

Furthermore, the research showed a notable association between the presence of MDR bacteria and the severity of the wound; here, 21 (80.76%) patients presented with MDR infections out of the 26 who had ulcers reaching the muscle or bone. This correlation signifies the potential for more complex and challenging treatment scenarios for MDR-infected wounds. For example, a study by Sun et al. about risk factors for lower extremity amputations demonstrates the correlation between the severity of the wounds, particularly grade 3–4, and the risk of amputation. Importantly, 50.3% of patients who underwent major amputations presented ulcers classified as Wagner 4 and 43.3%, Wagner 3 [28]. On this note, another statistically significant study finding is the positive correlation between the number of surgical procedures and MDR-infected wounds. This further supports the need for extensive treatment in cases of MDR infections.

Taking into account the statistically significant findings of this study, antibiotic intake before hospital admission may not only increase the risk of MDR colonization but also complicate the clinical course. More extensive wounds and more surgeries can affect the overall quality of life. Given that our protocol does not allow outpatient antibiotic treatment, the selection of MDR bacteria should decrease. Other authors have also mentioned prior antibiotic intake and extended hospital stays as risk factors for delayed wound healing. A study by Costa et al. noted that 22.3% of the pathogens they isolated were MDR. The mean hospital stay for patients infected by MDR was 79.8 days, compared to non-MDR patients (57.6 days). They concluded by underscoring the correlation between extended hospitalization and MDR infection [29].

Even more, complications such as wound dehiscence and graft rejection were more frequently observed in patients with MDR infections. An increased incidence of complications is to be expected because of the need for multiple surgical interventions and complex antibiotic treatment. Several studies also reported that MDR infections had high complication rates. A meta-analysis by Guo et al. [30], Ying et al. [31], Jing et al. [32] presents MDR infections as risk factors for DFU and high complications.

Our protocol states that all patients with bacterial infections must be admitted. This is to administer the correct local and antibiotic treatment, favoring proper wound healing. During their hospital stay, all patients receive antiseptic dressings for their ulcers. The dressings used are protective materials, such as silver nanoparticle-impregnated sheets and occlusive dressings impregnated with petrolatum and bismuth tribromophenate. 

One reconstructive method used for chronic ulcers is grafting. Silver nanoparticle-impregnated dressings combined with negative-pressure wound therapy have a symbiotic effect on wound healing and graft integration [33]. A study by de Francesco et al. states that hyaluronic acid salt 0.2% and collagenase ointment can reduce the total ulcer area and necrotic area with a higher wound recovery rate. However, this study states that this ointment is effective for small ulcers and home care contexts [34]. In another study, de Francesco et al. discuss the effectiveness of hyaluronic acid and silver sulfadiazine ointments on wound healing. This study mentions that surgical debridement can also be needed to reduce the microbial load [35]. While it is true that ointments with hyaluronic acid and collagenase or silver sulfadiazine can be effective for certain types of chronic wounds, they do not apply to those in our cohort. The patients included in our study were hospitalized with infected ulcers that required surgical debridement and more surgical procedures afterward. 

Furthermore, the average hospitalization time for patients with MDR-infected wounds was 25 days, in contrast to 10 days for those without. This significant difference once again highlights the profound clinical impact of MDR bacteria. In line with our observations, multiple authors suggest that MDR infections correlate with a prolonged hospital stay. Costa et al. noted an average of 79.8 days in MDR-infected patients, compared to 57.6, while in a systematic review, Sganga et al. propose a hospital stay longer than two weeks as a major risk factor for infection [29,36].

Another critical factor interfering with the process of wound healing is diabetes. Diabetes plays a pivotal role when discussing wound treatment, especially chronic wounds. Diabetic patients are considered high-risk patients due to the increased incidence and recurrence of diabetic foot ulcers (DFUs). The strongest predictor of DFU is a personal history of foot ulcers [37,38]. Research has shown that the high recurrence rate is due to precipitating factors. Factors that caused the ulcer in the first place (peripheral neuropathy, foot deformity, increased plantar stress, and peripheral vascular disease) are still present after the wound closure [39]. Although vascular disease can be improved with surgical procedures, profound peripheral neuropathy cannot [40]. Given the nature of the disease, glycemic levels, HbA1c, and BMI, the presence of peripheral neuropathy, vascular lesions, or foot deformities should be taken into account and considered aggravating factors [41,42]. This is another argument for a multidisciplinary approach and why we request specialty consultations for every patient.

Our analysis indicated a correlation between diabetic patients and ulcers located on the digits and feet, a widely recognized fact [43].

Moreover, multiple studies consider diabetic patients prone to infections—DFI (diabetic foot infections) as well as other community-acquired infections. In a study conducted by Yan et al., among 180 diabetic patients, 146 had positive bacterial cultures, and 84 were MDR [41,44]. Banu et al. conducted a prospective study where, out of 100 DFU patients, 82 had positive cultures with pathogens from the ESKAPE group [45]. Diabetic patients need a multidisciplinary approach since most of these patients present with multi-organ disease [46]. Our study showed that out of 82 diabetic patients, 56 presented with MDR infections. 

Furthermore, a lot of diabetic patients proved to need a higher number of surgical procedures compared to those without diabetes, with a mean difference of −0.67, thus underscoring the complexity and severity of wounds in this group of patients. An increased surgical need directly translates to a more extended hospitalization period. On average, diabetic patients had a 30-day hospital stay, which, compared to an average of 13 days for non-diabetic patients, is notably longer. This prolonged hospitalization time can also be attributed to a high rate of complications. In our sample, out of 82 diabetic patients, 16 suffered complications, compared to only 11 out of 117 non-diabetic patients. This disparity in complication rates further emphasizes how challenging the proper treatment of diabetic wounds can be. Aside from surgical complications, diabetic patients are susceptible to systemic complications as well, stressing the need for a multidisciplinary approach once again. Research on this matter is vast; a population-based study carried out by Iversen et al. states that the risk of death at - years for a diabetic patient who suffered from a diabetic ulcer is twice as high as the patients suffering from diabetes who did not develop an ulcer [47]. 

Last but not least, considering how chronic wounds can affect the quality of a patient’s life, new protocols must be implemented. By emphasizing the pivotal role of multidisciplinary approaches and reflecting on the challenges faced in treating chronic wounds, this study aims to highlight the main factors that interfere with the healing process of wounds. Nonetheless, the suggested protocol and conclusions discussed represent just a part of the research that still needs to be done. The protocol, as it stands, serves as a foundational core, and it can be adapted or expanded to address other challenges. This approach not only underscores our commitment to evolving patient care strategies but also to pioneering advancements in managing and treating complex wounds.

## 5. Conclusions

This study highlights the importance of introducing a chronic wound care protocol, especially when dealing with a rising number of MDR bacteria and complications. The analysis shows that such a protocol can more effectively address the multifaceted aspects of wound healing. As pointed out, a key summary of the protocol is to reduce the incidence of MDR bacteria and improve the patient’s quality of life. This means discharging patients with sterile, closed wounds.

In conclusion, our findings advocate for a multidisciplinary approach to chronic wound management. The protocol presented is a stepping stone to a better and more targeted treatment for chronic wounds.

## Figures and Tables

**Table 1:** Antibiotic resistance of the ESKASPE pathogens.

	*Enterococcus faecium*		*Staphylococcus aureus*		*Klebsiella pneumoniae*		*Acinetobacter baumanii*		*Pseudomonas aeruginosa*		*Enterobacter* spp.	
	nr (7)	%	nr (135)	%	nr (33)	%	nr (8)	%	nr (51)	%	nr (21)	%
Amoxicillin/ clavulanic acid	3	42.86	27	20.00	1	3.03	4	50.00	16	31.37	11	52.38
Oxacillin	1	14.29	27	20.00	1	3.03	3	37.50	6	11.76	3	14.29
Benzylpenicillin	2	28.57	64	47.41	4	12.12	3	37.50	7	13.73	4	19.05
Ticarcillin/ clavulanic acid	2	28.57	23	17.04	16	48.48	2	25.00	11	21.57	4	19.05
Piperacillin/ tazobactam	2	28.57	27	20.00	17	51.52	2	25.00	11	21.57	4	19.05
Cefuroxime	5	71.43	41	30.37	4	12.12	3	37.50	19	37.25	2	9.52
Ceftriaxone	4	57.14	29	21.48	3	9.09	2	25.00	16	31.37	2	9.52
Ceftazidime	1	14.29	14	10.37	4	12.12	2	25.00	7	13.73	3	14.29
Cefepime	1	14.29	12	8.89	3	9.09	2	25.00	5	9.80	4	19.05
Imipenem	0	0.00	9	6.67	2	6.06	2	25.00	10	19.61	0	0.00
Meropenem	0	0.00	4	2.96	1	3.03	2	25.00	6	11.76	0	0.00
Ciprofloxacin	4	57.14	31	22.96	7	21.21	3	37.50	13	25.49	7	33.33
Amikacin	0	0.00	4	2.96	1	3.03	1	12.50	1	1.96	0	0.00
Gentamicin	2	28.57	21	15.56	6	18.18	3	37.50	10	19.61	5	23.81
Tobramycin	0	0.00	15	11.11	5	15.15	2	25.00	6	11.76	1	4.76
Aztreonam	1	14.29	15	11.11	4	12.12	1	12.50	6	11.76	4	19.05
Streptomycin	2	28.57	5	3.70	2	6.06	1	12.50	3	5.88	1	4.76
Colistin	0	0.00	7	5.19	1	3.03	1	12.50	5	9.80	1	4.76
Trimethoprim/ sulfamethoxazole	1	14.29	14	10.37	5	15.15	2	25.00	7	13.73	6	28.57
Teicoplanin	1	14.29	3	2.22	0	0.00	0	0.00	2	3.92	1	4.76
Minocycline	0	0.00	7	5.19	3	9.09	1	12.50	1	1.96	1	4.76
Fosfomycin	0	0.00	3	2.22	0	0.00	1	12.50	3	5.88	0	0.00
Tetracycline	2	28.57	38	28.15	5	15.15	2	25.00	12	23.53	5	23.81
Vancomycin	1	14.29	2	1.48	0	0.00	0	0.00	1	1.96	0	0.00
Erythromycin	3	42.86	40	29.63	4	12.12	2	25.00	11	21.57	7	33.33
Clindamycin	1	14.29	36	26.67	2	6.06	1	12.50	4	7.84	3	14.29
Fusidic acid	0	0.00	8	5.93	0	0.00	0	0.00	3	5.88	1	4.76

**Table 2:** Relationship between antibiotic intake before hospitalization and positive MDR bacteria cultures. (Chi-square test, *p* = 0.035).

			MDR Bacteria		Total
			Absent	Present	
Antibiotic	Did not receive antibiotic	Count	51	71	122
		Expected count	43.7	78.3	122.0
	Did receive antibiotic	Count	25	65	90
		Expected count	32.3	57.7	90.0
Total		Count	76	136	212
		Expected count	76.0	136.0	212.0

**Table 3:** Relationship between the number of MDR bacteria, hospitalization time, and surgery number (Pearson correlation test—*p* < 0.001).

		**No. MDR Bacteria**	**Hospitalization (Days)**	**Surgery Number**
Number of MDR bacteria	Pearson correlation	1	0.464 **	0.347 **
	Significant (2-tailed)		<0.001	<0.001
	N	212	212	211
Hospitalization (days)	Pearson correlation	0.464 **	1	0.745 **
	Significant (2-tailed)	<0.001		<0.001
	N	212	212	211
Surgery number	Pearson correlation	0.347 **	0.745 **	1
	Significant (2-tailed)	<0.001	<0.001	
	N	211	211	211

** Correlation is significant at the 0.01 level (2-tailed).

**Table 4:** Wound site and diabetes evaluation (Chi-square test, *p* < 0.001).

		Site				Total
		Digits	Foot	Calf	Other	
Diabetes	Non-diabetic	14	21	39	56	130
	Diabetic	28	25	13	16	82
Total		42	46	52	72	212

## Data Availability

Data are contained within the article.
